# SPEDE‐sampler: An R Shiny application to assess how methodological choices and taxon sampling can affect Generalized Mixed Yule Coalescent output and interpretation

**DOI:** 10.1111/1755-0998.13591

**Published:** 2022-02-16

**Authors:** Clarke J. M. van Steenderen, Guy F. Sutton

**Affiliations:** ^1^ 59100 Department of Zoology and Entomology Centre for Biological Control Rhodes University Grahamstown/Makhanda South Africa

**Keywords:** barcoding, cryptic species, singletons, species delimitation

## Abstract

Species delimitation tools are vital to taxonomy and the discovery of new species. These tools can make use of genetic data to estimate species boundaries, where one of the most widely used methods is the Generalized Mixed Yule Coalescent (GMYC) model. Despite its popularity, a number of factors are known to influence the performance and resulting inferences of the GMYC. Moreover, the few studies that have assessed model performance to date have been predominantly based on simulated data sets, where model assumptions are not violated. Here, we present a user‐friendly R Shiny application, ‘SPEDE‐sampler’ (**SPE**cies **DE**limitation sampler), that assesses the effect of computational and methodological choices, in combination with sampling effects, on the GMYC model. Output phylogenies are used to test the effect that (1) sample size, (2) BEAST and GMYC parameters (e.g. prior settings, single vs multiple threshold, clock model), and (3) singletons have on GMYC output. Optional predefined grouping information (e.g. morphospecies/ecotypes) can be uploaded in order to compare it with GMYC species and estimate percentage match scores. Additionally, predefined groups that contribute to inflated species richness estimates are identified by SPEDE‐sampler, allowing for the further investigation of potential cryptic species or geographical substructuring in those groups. Merging by the GMYC is also recorded to identify where traditional taxonomy has overestimated species numbers. Four worked examples are provided to illustrate the functionality of the program's workflow, and the variation that can arise when applying the GMYC model to empirical data sets. The R Shiny program is available for download at https://github.com/clarkevansteenderen/spede_sampler_R.

## 1 INTRODUCTION

Taxonomy, including species delimitation and description, is vital to assessments of biodiversity (Delrieu‐Trottin et al., 2020; Inoue et al., 2020; Skaloud et al., 2015), conservation efforts (Devitt et al., 2019; Hosegood et al., 2020; Shirley et al., 2014), invasion biology (Boykin et al., 2012; Ross et al., 2010), biological control (Paterson et al., 2016; Peixoto et al., 2018) and predictions of the effects of climate change on species distributions and survival (Wang et al., 2019). Traditionally, taxonomists relied heavily on morphological variation to delimit, and later describe, new species. The development of molecular barcoding tools in the early 2000s (Hebert et al., 2003) has since provided an additional means of assessing species diversity and evolutionary history, which, in some cases, is a more robust approach (Packer et al., 2009). The identification of cryptic species (Paterson et al., 2016) and immature life stages (Shin et al., 2015), for example, is often an impossible task using morphology alone. Molecular tools have accelerated species discovery (e.g. Mutanen et al. (2013) and references therein), with nearly 10,000 publications containing the keywords ‘DNA barcoding’ since 2003 (PubMed NCBI search ( https://www.ncbi.nlm.nih.gov/pubmed/ )).

A variety of species delimitation algorithms have been developed to estimate species boundaries from DNA barcodes, where distinct groups are most frequently referred to as ‘molecular operational taxonomic units’ (MOTUs), evolutionarily significant units (ESUs), ‘genospecies’, ‘phylospecies’, ‘phylotypes’ or ‘recognizable taxonomic units’ (RTUs; Fontaneto et al., 2015; Luo et al., 2018; Wiens, 2007). These groups are hypothesized species that require further exploration. Numerous delimitation methods have been developed, which utilize either (1) phylogenetic trees, (2) allele‐sharing data or (3) genetic distance matrices to estimate species boundaries (Dellicour & Flot, 2018; Flot, 2015). Popular methods include Automatic Barcode Gap Discovery (ABGD; Puillandre et al., 2012), Generalized Mixed Yule Coalescent (GMYC; Fontaneto et al., 2007; Fujisawa & Barraclough, 2013; Pons et al., 2006), Poisson tree processes (PTP/bPTP; Zhang et al., 2013), multirate PTP (mPTP; Kapli et al., 2017) and Bayesian phylogenetics and phylogeography (BPP; Yang, 2015).

The GMYC model is a widely applied ultrametric tree‐based tool for species delimitation that implements maximum‐likelihood statistics to single‐locus genetic data (predominantly mitochondrial; Fontaneto et al., 2007; Fujisawa & Barraclough, 2013; Pons et al., 2006). The model assesses when branching rates in an ultrametric phylogeny transition from the species (interspecific) to the population (intraspecific processes) level. In this way, genetic cluster groups are separated by longer internal branch lengths (Fujisawa & Barraclough, 2013). Model assumptions include that (1) species are monophyletic, (2) there is no intraspecific geographical structuring, and (3) there is no extinction (Fujisawa & Barraclough, 2013). The method has become very popular in ecology because it does not require prior knowledge of the target study group, which makes it a particularly useful tool for studies involving species for which taxonomic knowledge is limited or non‐existent (Talavera et al., 2013).

Generalized Mixed Yule Coalescent performance (and thus species discovery), however, has been shown to be affected by a number of methodological and computation factors (Blair and Bryson Jr, 2017; Dellicour & Flot, 2015; Esselstyn et al., 2012; Fonseca et al., 2021; Hamilton et al., 2014; Magoga et al., 2021; Tang et al., 2014). The GMYC method is subject to lower performance when there are few species (O'Meara, 2010), singletons (species represented by one individual that can result in overestimations of species numbers; Fujisawa & Barraclough, 2013), and/or recent, rapid divergences (Reid & Carstens, 2012). Species numbers may regularly be overestimated due to the sensitivity of delimitation algorithms to intraspecific population structure, which can be exacerbated by incomplete sampling (Papadopoulou et al., 2009; Sukumaran & Knowles, 2017). In the *Aphonopelma* tarantula genus, for example, Hamilton et al. (2014) found that the number of GMYC species varied ‘alarmingly’ due to incomplete or biased sampling. Instead of improved performance with greater sampling, the authors found larger variation in species richness estimates. Tang et al. (2014) highlighted the effect that branch smoothing (correcting for rate heterogeneity so that clock‐like, ultrametric phylogenies are produced) can have on the aberrant lumping or splitting of groups due to variability in branch lengths, and how this can drastically alter inferences made. In another example involving *Hipposideros* bats, Esselstyn et al. (2012) found that the accuracy and precision of the GMYC method declined when effective population size (Ne) and speciation rate (i.e. rapid divergence) increased.

The performance of the GMYC model has been predominantly tested on simulated data where the effects of factors are controlled, and the model's assumptions are not violated (Esselstyn et al., 2012; Fujisawa & Barraclough, 2013; Papadopoulou et al., 2009; Talavera et al., 2013). Most applications of the GMYC using empirical data will, however, likely violate these assumptions. This highlights a large knowledge gap in its performance when using data sets that have unknown species boundaries, are subject to undersampling bias and unequal sampling effort, or a combination of these factors. Fonseca et al. (2021), for example, have recently developed an R package to assess the statistical fit of the GMYC model to data sets for which there are an unknown number of putative species.

Here, we present ‘SPEDE‐sampler’, a user‐friendly R Shiny application that assesses the effects of sample size and singletons, in combination with different BEAST (Bouckaert et al., 2019) and GMYC parameter settings, on species delimitation using the GMYC model (Figure S1). This software can help users of the GMYC method to assess limitations arising from their data, highlight potential undiscovered diversity and interpret GMYC output in a biologically meaningful way. This manuscript details the functionality of the application through the use of four worked examples: (1) mitochondrial 12S rRNA sequence data derived from cochineal insects (Hemiptera: Dactylopiidae; van Steenderen et al., 2021), (2) mitochondrial COI data from a DNA barcoding study of Congolese and Lower Guinean fishes (Sonet et al., 2019), (3) COI data from tachinid flies (Smith et al., 2006) and (4) COI data from Madagascan ants (Smith et al., 2005). The R Shiny SPEDE‐sampler application is freely available on GitHub with installation instructions and a user guide with a fully worked example.

## 2 FUNCTIONALITY OVERVIEW

### 2.1 R Shiny application

Installation instructions are in the README document in the SPEDE‐sampler GitHub repository. The workflow begins with the uploading of an aligned multiple sequence alignment (MSA) file that is subsetted and then randomly resampled a desired number of times without replacement (Figure S1 steps 1 and 2). For example, a MSA file of 500 sequences might be uploaded, randomly subsetted to 50% of the data, and repeated 10 times. This will yield 10 FASTA files comprising a random assortment of 250 sequences in each. The user has the option of uploading an Excel.CSV file containing predefined grouping information for each sequence, which can be used to ensure that at least one representative sequence for each predefined group is included in each resampled file. Each of these FASTA files is then used to generate an .XML file for analysis in BEAST (Bouckaert et al., 2019), using the R package ‘beautier’ (Bilderbeek & Etienne, 2018; Figure S1 step 3). The user can set up the .XML file in the SPEDE‐sampler application, with the option of selecting a site and clock model, clock rate, tree prior, associated rate distributions and an MCMC value. For large MSA files, it is advisable to run BEAST on the CIPRES Science Gateway platform ( http://www.phylo.org/ ) for faster performance. The resulting .TREES files produced by BEAST need to be inputted to TreeAnnotator in order to obtain maximum clade credibility (MCC) trees. The user can set a percentage burn‐in and select from different node height options. The resulting MCC trees are then used as input for GMYC analyses (Figure S1 steps 4–9). Tracer is available via the ‘tracerer’ R package to check effective sample size (ESS) scores and for MCMC convergence. LogCombiner is available in SPEDE‐sampler as an optional means of reducing the size of the .TREES files by resampling states at a lower frequency.

The user can optionally upload a .CSV file containing morphospecies, ecotypes, or other relevant predefined grouping information for each sequence in the BEAST‐generated phylogenies. The GMYC method does not require prior grouping information, but this feature is available in SPEDE‐sampler in order to compare DNA‐based species delimitation to traditional taxonomy. The GMYC species estimates are compared with these predefined groups in order to assess a match rate, and to what degree groups have been ‘oversplit’ by the GMYC method. Comparing DNA‐based GMYC estimates to existing morphologically or ecologically defined species in this way can be very useful in deciding whether the taxonomy is likely outdated and contains possible cryptic species.

The user can choose between a single approach (Pons et al., 2006) and multiple GMYC threshold (Monaghan et al., 2009) approach. Applying a multiple threshold method may be useful in large data sets where there is significant variation in intra‐ and interspecific genetic divergences. Generally, however, a single‐threshold approach is recommended as it is less likely to oversplit (Blair and Bryson Jr, 2017; Fujisawa & Barraclough, 2013; Talavera et al., 2013).

Once the GMYC analysis is complete for all BEAST tree files, the application records the estimated number of entities and clusters (the number of delimited groups comprising two or more samples, including and excluding singleton sequences, respectively), and optionally compares the match rate of user‐defined groups to estimated GMYC species groups. Additionally, the application assesses (1) the effect that singletons have on species delimitation, (2) the number of GMYC merges and exact matches and (3) the manner in which the GMYC method splits species relative to predefined groups. The term ‘oversplit’, as used here, does not necessarily always imply the incorrect splitting into too many species, but rather that the splitting ratios highlight which species groups may contain potential undiscovered biodiversity. The term can be synonymous with ‘discordant splitting’. Box 1 provides the terms used in this manuscript and their definitions.

BOX 1  

**Clusters** The number of delimited groups comprising two or more samples, excluding singletons.

**Entities** The number of delimited groups comprising two or more samples, including singletons.

**Exact match** An instance during scoring when all the samples belonging to a particular user‐defined group (morphospecies or other user‐defined group) correspond to the same GMYC species.

**Split match** An instance during scoring when the samples belonging to a particular user‐defined group (morphospecies or other user‐defined group) are split into two or more GMYC species groups. This indicates the possibility of the underestimation of species richness by the user.

**Match (y/n)** A means of denoting, in the work‐through of the R code, whether each GMYC species comprises one unanimous user‐predefined group.

**Merge** An instance during scoring when two or more user‐defined groups are merged into one GMYC species. This indicates the possibility of an overestimation of species richness by the user.

**Oversplitting** The outcome where the GMYC model has estimated more species than those estimated by the user (=‘discordant splitting’). This could mean either (1) the incorrect splitting into too many species, or (2) the genuine presence of undiscovered biodiversity or cryptic species.

**Undersplitting** The outcome where the GMYC model has estimated fewer species than those estimated by the user. This could mean either (1) the incorrect merging into too few species or (2) the genuine presence of lower biodiversity than expected (e.g. variations in intraspecific morphological characters that are mistaken for being interspecific).

**Splitting ratio** The ratio of the total number of estimated GMYC species to the total number of user‐defined groups in the data set. A value greater than 1 indicates oversplitting, while a value less than 1 denotes undersplitting. A value of 1 means perfect agreement between the GMYC and the user’s estimates.

**(Overall) percentage match** The overall proportion of successful matches (records of ‘y’) in a data set. This includes cases of both exact matches and split matches, and is calculated with and without singletons.

**Singleton** A species represented by only one individual/genetic sequence.

#### 2.1.1 Calculation of GMYC metrics

**FIGURE 1:**
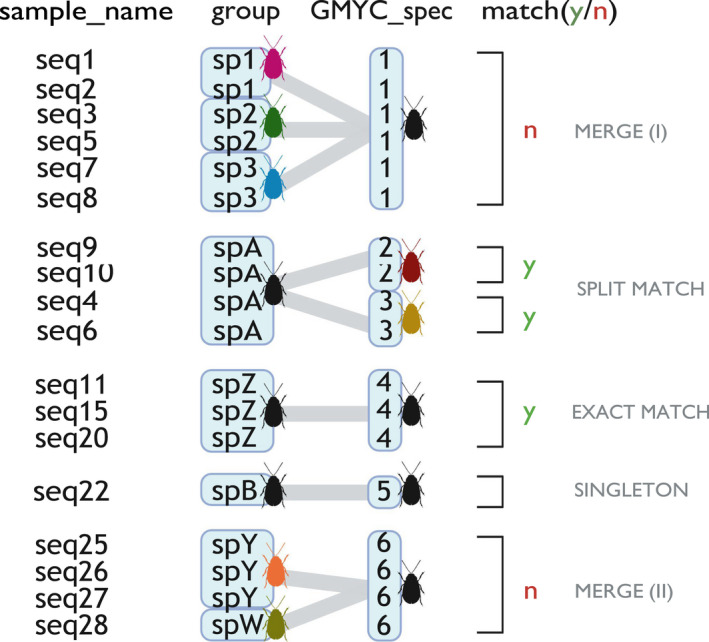
Detailed diagrammatic explanation of how SPEDE‐sampler determines cases of merges, splits, exact matches and singletons in a hypothetical example of one GMYC analysis (i.e. one BEAST phylogeny input). A merge occurs when the user has overestimated the number of groups, and the GMYC has lumped them into one (merge type I). A merge is recorded even if one group is a singleton (e.g. spW in merge type II). A match can take two forms: (1) a split, where the user has underestimated the number of groups, or (2) an exact match, where a user‐defined group and a GMYC species delimitation corroborate exactly. Singletons occur when there is one sequence representing a GMYC species. Figure created with BioRender.com

The R Shiny application generates a summary table of each sample name, its designated GMYC species group number, the corresponding user‐predefined group, and a score of ‘y’ (yes) or ‘n’ (no) to denote whether the GMYC species designations and the user's predefined groups consistently match (Figure S1 and Figure 1). In a similar approach to Magoga et al. (2021), an ‘n’ outcome is recorded as a ‘merge’, where the GMYC has lumped two or more groups defined by the user (i.e. the user has overestimated species richness) (‘merge type I’). A merge is recorded even if one or more of these groups is represented by a singleton (‘merge type II’). A ‘y’/match outcome can take two forms, namely (1) ‘split’, where the GMYC has split one user‐defined group into two or more groups (i.e. the user has underestimated species richness), or (2) ‘exact’, where the user‐defined groups match the GMYC estimates exactly (Figure 1). Overall percentage match scores, including (*m*_i_) and excluding (*m*_e_) singletons, are subsequently calculated as shown in the following equations.

$$m_{i}=\frac{\Sigma y+\Sigma \text{singletons}}{\Sigma y+\Sigma n+\Sigma \text{singletons}}\times 100$$

$$m_{e}=\frac{\Sigma y}{\Sigma y+\Sigma n}\times 100$$

Overall percentage match scores include both match scenarios (i.e. ‘split’ and ‘exact’ matches), which is different from the exact match score calculation. Exact match scores are calculated per group (i.e. morphospecies or other user‐defined group) as the number of times that a particular group is scored as an exact match, averaged across all GMYC runs. The splitting ratio is calculated as the ratio of the estimated number of GMYC species (including and excluding singletons) to the number of user‐predefined groups, and the percentage of singletons is calculated as the ratio of the sum of the number of singletons to the number of GMYC species. Box 2 provides a hypothetical example of how SPEDE‐sampler calculates these metrics in practice, using the scenario shown in Figure 1.

The user can explore and download a variety of summary plot outputs, including the fluctuations in the number of clusters (excluding singletons) and entities (including singletons) across tree iterations, boxplots for the overall number of clusters and entities across all iterations, particular input trees with GMYC support values, changes in percentage matches across tree iterations, and boxplots and barplots for groups that were oversplit or merged. Accumulation curves show the number of clusters and entities at each sample size with a 95% confidence interval band using the replicated data sets. This is fitted with the geom smooth() function in the ggplot2 package. Each plot can be downloaded in .PNG, .SVG, or .PDF format, with customizable dimensions and resolutions where applicable.

BOX 2  

Using Figure 1 as an example of the output of one GMYC analysis:

There are six GMYC species and 8 user‐defined groups.

1. Species 1 (sp1), 2 (sp2) and 3 (sp3) would be recorded as a merge (**merge type I**), while species A (spA) would be flagged as being oversplit by a factor of 2 by the GMYC model. Species A may present a case of two previously undiscovered cryptic species, or merely intraspecific population structuring. Species Z (spZ) would be recorded as an exact match. In a hypothetical scenario, if a total of three GMYC analyses were run, where species Z was recorded as an exact match in two of the runs, then Species Z would have a 67% **exact match** score (i.e. exact match score = Σ(exact match count)/(the number of input files) x 100 = 2/3 x 100 = 67%).
2. Overall, with species Z being the only user‐predefined group with an exact GMYC match, the **exact match incidence** in this GMYC run = (the number of user‐defined groups with a recorded exact match)/(the number of user‐defined groups) = 1/8 = 13%.
3. Species B (spB) would be recorded as a singleton.
4. Species Y (spY) and species W (spW) would be recorded as a merge, even though spW is a singleton (**merge type II**). The user should pay attention to these cases, as they might be potential taxonomic misidentifications.
5. The **splitting ratio** including singletons would be calculated as: (the number of GMYC species)/(the number of user‐defined groups) = 6/8 = 0.75. The splitting ratio excluding singletons would be: (the number of GMYC species ‐ the number of singletons)/(the number of user‐defined groups) = (6‐1)/8 = 0.63. Splitting ratios < 1 indicate that there is a high incidence of overall merging by the GMYC due to an overestimation of species richness by the user. Splitting ratios > 1 indicate that species richness has been underestimated by the user and that there may be cryptic species in the mix. Ratios that equal 1 indicate that the number of user‐defined groups and the number of GMYC species are the same.
6. The overall **percentage match**, including singletons $\begin{array}{c} \left(m_{i}\right)=\sum \left(y\right)+\sum \left(\text{singletons}\right)/\left(\sum \left(y\right)+\sum \left(n\right)+\sum \left(\text{singletons}\right)\right) \end{array}$ $\begin{array}{c} =\left(3+1\right)/\left(3+2+1\right)=67\% \end{array}$. Excluding singletons, the percentage match $\left(m_{e}\right)=\sum \left(y\right)/\left(\sum \left(y\right)+\sum \left(n\right)\right)=3/\left(3+2\right)=60\%$. In this case, singletons are causing a 7% inflated percentage match estimate.
7. The **percentage of singletons** $=\sum \left(\text{singletons}\right)/\left(\text{number of GMYC species}\right)=1/6=17\%$.

## 3 WORKED EXAMPLES USING EMPIRICAL DATA SETS

### 3.1 Methods

To illustrate the functionality of SPEDE‐sampler, we present four worked examples listed below. A step‐by‐step work‐through for the cochineal data set is available on the GitHub repository. All the relevant data files are available for download.

#### 3.1.1 Cochineal 12S data

The Dactylopiidae are a monogeneric group that feed exclusively on cacti (De Lotto, 1974). There are currently 11 described species and multiple intraspecific lineages that are frequently used as biological control agents of invasive cactus species (Winston et al., 2014). Mitochondrial 12S rDNA (*n* = 142, 386 nucleotide bases) genetic sequences from van Steenderen et al. (2021) were used for our first worked example (GenBank Accession nos MN219994‐MN220135). Ecospecies (=ecotype) assignments were based on the host plants from which the specimens were collected, as species and intraspecific lineages are host‐specific. Host specificity is usually restricted to a particular cactus genus or closely related genera (De Lotto, 1974). There were five predefined ecotypes in this data set. Additionally, there were six known intraspecific lineages within *Dactylopius tomentosus*, but these were not set as predefined groups to test whether SPEDE‐sampler would detect them. It is currently accepted that these entities are intraspecific lineages based on interbreeding trials, although there may be cases of cryptic or sibling species in this group (Mathenge et al., 2010).

#### 3.1.2 Tachinid fly COI data

Tachinids (Diptera: Tachinidae) are one of the most species‐rich fly families, comprising close to 10,000 described species globally (Stireman et al., 2006). Tachinid larvae are endoparasitoids of insects and other arthropods, and appear to be more host‐specific than previously believed (Janzen & Hallwachs, 2021). This is an important factor in terms of their use in biological control programmes targeting insect pests (e.g. the gypsy moth, *Lymantria dispar* (Lee & Pemberton, 2019)). Smith et al. (2006) conducted a DNA barcoding study to assess species richness and host specificity within the *Belvosia* Robineau–Desvoidy genus. The authors had 20 morphospecies identified by an expert taxonomist, and, after COI barcoding, discovered a further 12. They concluded that the group contained a suite of host‐specific cryptic species. We used these COI sequences (GenBank Accession nos DQ3480895–DQ348780, *n* = 736, 668 base pairs) as a second worked example.

#### 3.1.3 Congolese and Guinean fish COI data

Sonet et al. (2019) undertook a barcoding study of fishes collected from the Middle and Lower Congo River and three drainage basins in the Lower Guinean provinces of Kouilou–Niari, Nyanga and Ogowe. The Congo basin is the second largest catchment area in the world and is a biodiversity hot spot that is still largely undersampled (Thieme et al., 2005). Sonet et al. (2019) recorded 194 morphospecies (55 of which were singletons) and reported at least 17 putative new species based on their genetic results. Their COI sequences (GenBank Accession nos MK073961‐MK074701, *n* = 741, 652 base pairs) were used as our third worked example.

#### 3.1.4 Madagascan ants COI data

An estimated 96% of the ~1000 ant species in Madagascar are endemic, where approximately 75% are undescribed (Fisher, 1997). Despite being declared a biodiversity hot spot, the island's arthropod fauna are under threat of extinction in the face of habitat destruction and invasive species (Rabearivony et al., 2010). Assessing species richness and prioritising protected areas is a vital task in conservation planning. Smith et al. (2005) generated a COI barcode database of 267 ant specimens (GenBank Accession nos DQ176049–DQ176316, 662 base pairs) collected in northeastern Madagascar. The authors recorded 88 morphospecies, and between 117 and 126 MOTUs based on their genetic analyses. We use their data set as a fourth worked example.

#### 3.1.5 Generalized Mixed Yule Coalescent analysis

The multiple sequence alignment files in each case study (Supporting information) were uploaded to SPEDE‐sampler in independent analyses, where they were first randomly resampled 10 times, without replacement, for subsets of 25%, 50%, 75%, and 100% of the sequence data. A random seed was set for each resampling event. The resampled files were subsequently used as input for the creation of .XML files, where the following parameters were implemented: GTR site model, strict clock (clock rate = 1), Yule tree prior with a uniform birth rate distribution, and the MCMC set to 5 million. The resulting .XML files were loaded into BEAST2 and run with BEAGLE (Ayres et al., 2012). The CIPRES Science Gateway portal was used to run BEAST2 for the tachinid and fish data sets that had >700 sequences.

The LOG files generated by BEAST2 were uploaded to Tracer to check for convergence. TREES files were uploaded to TreeAnnotator, where burn‐in was set to 25%, and heights to ‘median’. The resulting .NEX files for each Bayesian tree were then used as input for single‐threshold GMYC analyses, where a random seed was set. A .CSV Excel file containing the predefined groups and associated name for each sequence was uploaded in order to compare the output of the GMYC model to the predefined grouping information. All results were stored, and .CSV data files were amalgamated across data subsets for each statistic, and subsequently plotted.

### 3.2 Results and discussion

#### 3.2.1 Cochineal insects

**FIGURE 2:**
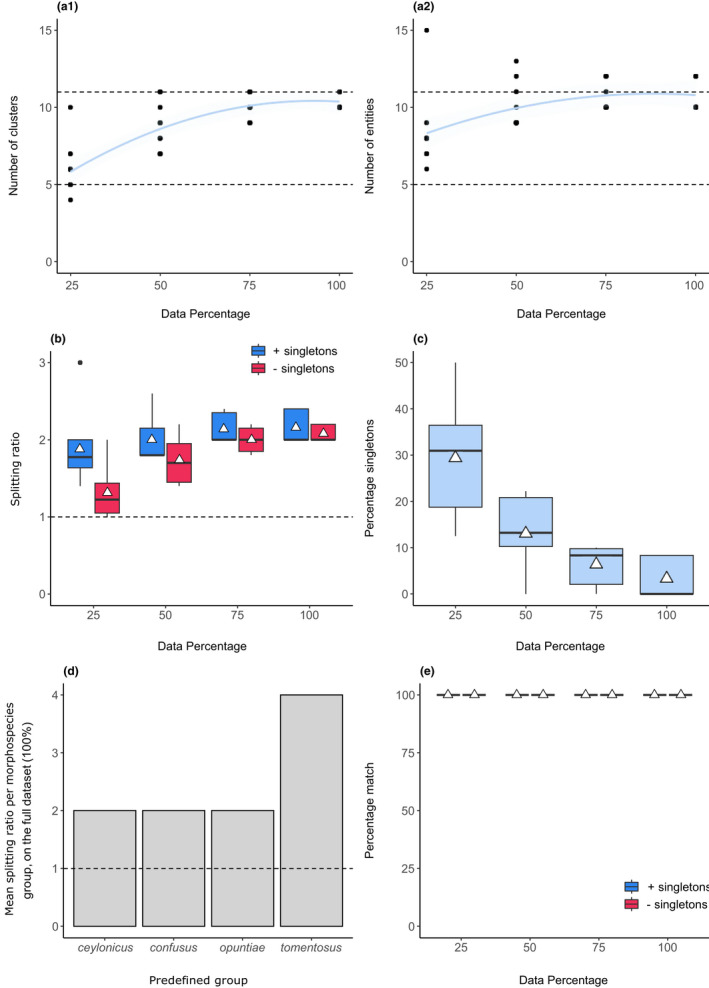
SPEDE‐sampler results for 142 12S cochineal sequences. (a1 and a2) The number of clusters and entities across subsetted data set sizes. The light blue band represents a 95% confidence interval. The dotted lines at *y* = 5 and *y* = 11 are the number of predefined ecotypes, and known ecotypes plus intraspecific lineages, respectively. (b) A boxplot of the splitting ratios across data set sizes, including (blue) and excluding (red) singletons. The dotted line at y = 1 indicates the expected ratio if no splitting occurred. (c) A boxplot of the percentage of singletons across data set sizes. (d) The mean splitting ratios of predefined ecotypes that exceeded a ratio of 1 (dotted horizontal line). (e) A boxplot of the percentage matches between predefined ecotypes and GMYC species, including (blue) and excluding (red) singletons. White triangles represent means

We found an average of 10.4 ± 0.52 and 10.8 ± 1.03 clusters and entities, respectively, in the full data set (100%; Figure 2a1,a2). This aligned with the expected number of ecotype and intraspecific lineages (*n* = 11). The asymptotic pattern in the curves in Figure 2a1,a2 suggests that adding more specimens from the sampling sites in the study is unlikely to yield greater species richness.

Average splitting ratios exceeding a value of 1 (Figure 2b; i.e. the ratio of the number of GMYC species estimates to predefined ecotypes) for all data sizes indicated that the number of predefined ecotypes (*n* = 5) was an underestimate of the diversity present. This was expected, as the intraspecific lineages in *D*. *tomentosus* were deliberately not divided into ecotypes, as discussed previously. The splitting ratio tended to be higher when singletons were included. This was most pronounced in the 25% data set, where the average percentage of singletons was 29.4% ± 12.5. The average percentage of singletons present dropped to 3.33% ± 4.3 in the full data set (Figure 2c). We did not record any GMYC merges in the full data set, and found two 269 cases of exact matches (40% of user‐defined ecospecies), namely *D*. *austrinus* and *D*. *opuntiae*, 270 with mean exact match scores of 100% and 60%, respectively.

Four ecotypes were identified as containing greater diversity than expected, namely *D*. *ceylonicus*, *D*. *confusus*, *D*. *opuntiae* and *D*. *tomentosus* (Figure 2d). van Steenderen et al. (2021) did find two strongly supported *D*. *ceylonicus* clades representing specimens collected in South Africa and Australia. Similarly, *D*. *opuntiae* and *D*. *confusus* specimens were collected across a wide geographical range and from different host plants. This pattern of intraspecific structuring could be misinterpreted as species‐level divergences by the GMYC model. *Dactylopius tomentosus* displayed the highest mean splitting ratio, indicating a fourfold underestimate of diversity. This corroborates with van Steenderen et al. (2021), who found four strongly supported intraspecific lineages within this species, namely ‘imbricata’, ‘californica’, [‘echinocarpa x acanthocarpa’, ‘bigelovii’, ‘cylindropuntia’], and ‘cholla’. The percentage match scores between predefined ecotypes and GMYC species delimitations were 100% across all data set sizes, irrespective of the inclusion of singletons (Figure 2e).

#### 3.2.2 Tachinid flies

**FIGURE 3:**
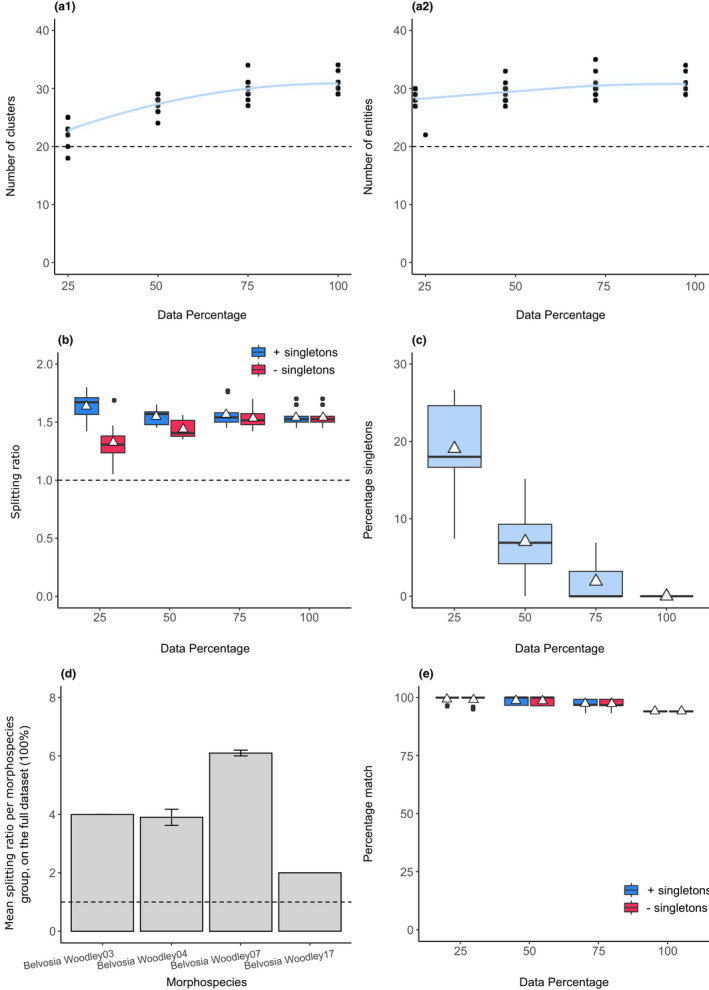
SPEDE‐sampler results for 736 COI tachinid sequences. (a1 and a2) The number of clusters and entities across subsetted data set sizes. The light blue band represents a 95% confidence interval. The dotted lines at y = 20 is the number of predefined morphospecies. (b) A boxplot of the splitting ratios across data set sizes, including (blue) and excluding (red) singletons. The dotted line at *y* = 1 indicates the expected ratio if no splitting occurred. (c) A boxplot of the percentage of singletons across dataset sizes. (d) The mean splitting ratios of morphospecies groups that exceeded a ratio of 1 (dotted horizontal line). (e) A boxplot of the percentage matches between predefined morphospecies and GMYC species, including (blue) and excluding (red) singletons. White triangles represent means

We found an average of 30.8 ± 1.62 for both the number of clusters and entities in the full data set (100%; Figure 3a1,a2). These measures are the same because there were no singletons recorded (clusters and entities are the same except for the inclusion of singletons in the measure for entities). This corroborates the results of Smith et al. (2006), who reported 32 genetic species clusters, but is approximately 1.6‐fold more than the number estimated by morphological taxonomy (*n* = 20). As was the case with the cochineal insects, the asymptotic lines in Figure 3a1,a2 suggest that the addition of more tachinid specimens from the sampling sites in the study is unlikely to yield greater species richness estimates.

Mean splitting ratios exceeded a value of 1 across all data set sizes (Figure 3b), with an average of 1.54 ± 0.08 in the full data set (100%), both including and excluding singletons. This indicated again that the number of predefined morphospecies underestimated the species diversity in the data set. The diversity of four morphospecies was underestimated, namely *Belvosia* Woodley03, *Belvosia* Woodley04, *Belvosia* Woodley07 and *Belvosia* Woodley17, with splitting ratios of 4, 3.9 ± 0.88, 6.1 ± 0.32, and 2, respectively (Figure 3d). Smith et al. (2006) report three species within *Belvosia* Woodley03 (one less than our estimate), four species within *Belvosia* Woodley04 (corroborating our result) and eight species within *Belvosia* Woodley07 (two more than our estimate). The authors report only one MOTU for *Belvosia* Woodley17, while our results suggest that there may be two. As was reported in the cochineal example, some lineages displayed intraspecific structuring that could be mistaken for species‐level divergence. The nine *Belvosia* Woodley17 COI samples (all sharing the same host noctuid *Pseudaletia sequax*) were split into two groups: [DQ348799, DQ348800, DQ348801, DQ348802, DQ348805, DQ348806], and [DQ348803, DQ348804, DQ348807]. It is possible that this is due to a sequencing artefact, as four of these sequences (DQ348799, DQ348801, DQ348803 and DQ348804) comprised approximately 44% ambiguous (*N*) base pairs.

We found only two cases of merging (10% of user‐defined morphospecies) in the full data set, namely *Belvosia* Woodley01, sequence DQ348107, that the GMYC grouped with *Belvosia* Woodley02 samples, and *Belvosia* Woodley12, sequence DQ348776, that the GMYC grouped with *Belvosia* Woodley11 samples. We found 15 cases of exact matches (75% of user‐defined morphospecies; Data S7).

The presence of singletons did not appear to affect percentage match scores across data sizes, where values always exceeded at least 93% (Figure 3e).

#### 3.2.3 Congolese and Guinean fishes

**FIGURE 4:**
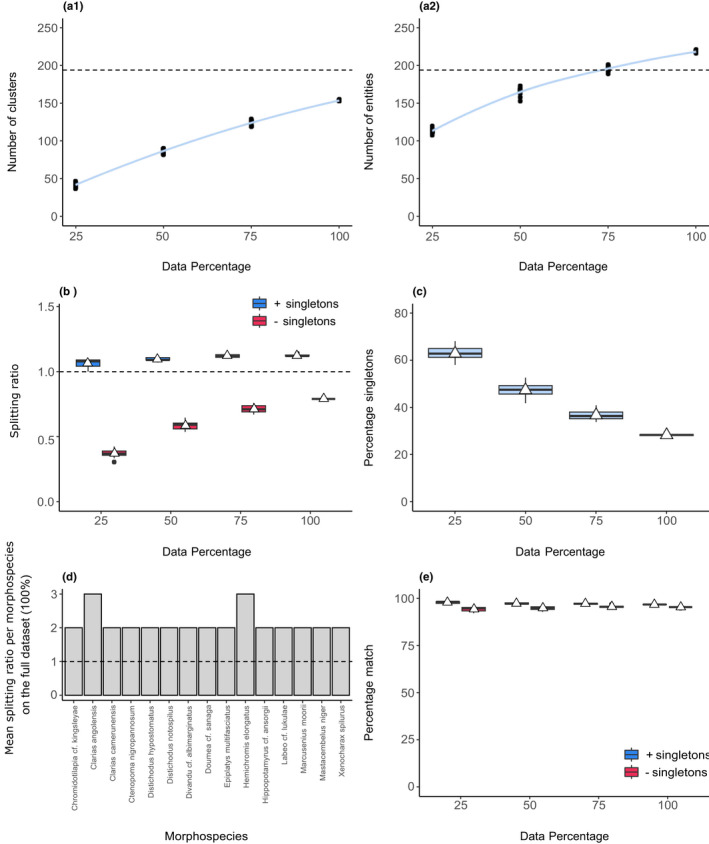
SPEDE‐sampler results for 741 COI fish sequences. (a1 and a2) The number of clusters and entities across subsetted data set sizes. The light blue band represents a 95% confidence interval. The dotted lines at y = 194 is the number of predefined morphospecies. (b) A boxplot of the splitting ratios across dataset sizes, including (blue) and excluding (red) singletons. The dotted line at *y* = 1 indicates the expected ratio if no splitting occurred. (c) A boxplot of the percentage of singletons across data set sizes. (d) The mean splitting ratios of morphospecies groups that exceeded a ratio of 1 (dotted horizontal line). (e) A boxplot of the percentage matches between predefined morphospecies and GMYC species, including (blue) and excluding (red) singletons. White triangles represent means

We report an average of 153.4 ± 0.7 and 218.1 ± 1.52 clusters and entities, respectively, for the full data set (100%; Figure 4a1,a2). This is within the same range as the results reported by Sonet et al. (2019), at 194 morphospecies. Only when singletons were included did the average splitting ratios exceed a value of 1 across all data sizes (with an average of 1.12 ± 0.01 in the full data set (100%); Figure 4b). The exclusion of singletons led to merging by the GMYC (splitting ratios < 1). In the full data set, we found seven cases of GMYC merges (4% of user‐defined morphospecies; Data S8). Notably, the GMYC merged the morphospecies *Coptodon congicus* and *Coptodon tholloni*; *Ctenopoma ocellatum*, *Ctenopoma* sp. Lefini, *Ctenopoma cf*. *maculatum* and *Ctenopoma acutirostre*; and *Labeobarbus sp*. *intermediate* and *Labeobarbus sp*. *inkisi*.

The trajectory of the curves in Figure 4a1,a2 suggest that the addition of more sequences is likely to yield increased species richness estimates, as would be expected in this poorly sampled region.

The percentage of singletons remained high across data sizes (Figure 4c), and resulted in the discrepancy between the splitting ratios between the inclusion and exclusion of singletons seen in Figure 4b. We identified 15 morphospecies for which diversity may have been underestimated, particularly *Clarias angolensis* and *Hemichromis elongatus* (Figure 4d). Sonet et al. (2019) did report that *Clarias angolensis* comprised at least two haplogroups and that *Hemichromis elongatus* comprised four barcode clusters. Percentage match scores between morphospecies and GMYC estimates remained above 90% across data set sizes, with and without singletons (Figure 4e). The authors reported that 92.8% of their morphospecies assignments corresponded to species clusters based on their barcoding results. We report similar percentage match estimates on the full data set, at 96.74 ± 0.13 and 95.37 ± 0.19% including and excluding singletons, respectively (Figure 4e). However, we found only 116 cases (60% of user‐defined morphospecies) of exact matches (Data S9).

#### 3.2.4 Madagascan ants

**FIGURE 5:**
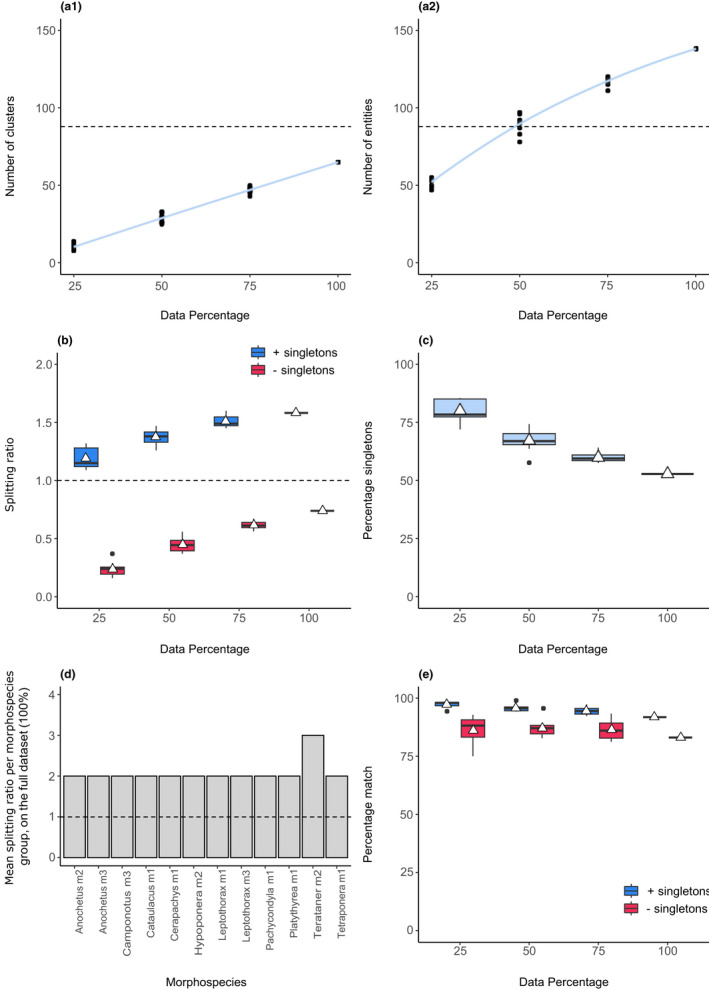
SPEDE‐sampler results for 267 COI ant sequences. (a1 and a2) The number of clusters and entities across subsetted dataset sizes. The light blue band represents a 95% confidence interval. The dotted lines at *y* = 88 is the number of predefined morphospecies. (b) A boxplot of the splitting ratios across dataset sizes, including (blue) and excluding (red) singletons. The dotted line at y = 1 indicates the expected ratio if no splitting occurred. (c) A boxplot of the percentage of singletons across dataset sizes. (d) The mean splitting ratios of morphospecies groups that exceeded a ratio of 1 (dotted horizontal line). (e) A boxplot of the percentage matches between predefined morphospecies and GMYC species, including (blue) and excluding (red) singletons. White triangles represent means

We found an average of 65 and 138 clusters and entities, respectively, on the full data set (100%; Figure 5a1,a2). This is within the range of the 88 morphospecies recorded by Smith et al. (2005), but it appears that the high incidence of singletons in the data set may have contributed to species richness overestimates (Figure 5b,c), with an average splitting ratio of 1.57 in the full data set when the percentage of singletons was 52.9%. As was the case in the fish example, the absence of singletons led to GMYC lumping across all data set sizes (Figure 5a1,b).

Our results indicated that diversity had been potentially underestimated in 12 morphospecies (Figure 5d), particularly *Terataner* m2 with a splitting ratio of 3. Upon closer inspection of the collection sites for *Terataner* m2 specimens, it is more likely that this is a result of intraspecific geographical structuring. In the full data set, we found seven cases (8% of user‐defined morphospecies) of GMYC merges (Data S10) and 29 cases (33% of user‐defined morphospecies) of exact matches (Data S11), where all cases had mean exact match scores of 100%.

The presence of singletons resulted in decreased percentage match scores, which dropped by nearly 9% when singletons were excluded in the full data set (Figure 5e). This is the lowest reported percentage match score across case studies, at 83.08%.

The trajectories of the lines in Figure 5a1,a2 suggest that species richness estimates are likely to increase with greater sampling effort. This is expected given the high incidence of undescribed ant diversity in the northeastern Madagascan region.

### 3.3 Case study summary

#### 3.3.1 Sample size and population structure

Across all four case studies presented here, we found that increased taxon sampling (1) reduced the percentage of singletons present, (2) tended to result in higher splitting ratios and (3) did not negatively affect percentage match scores between GMYC species and predefined groups. The number of clusters tended to approach the number of predefined groups as sample size increased, with the exception of the tachinid flies where these values were overestimated across all data set sizes, and the ants where the number of entities far exceeded expected values due to the high incidence of singletons. Overall, species richness estimates (both clusters and entities) did not vary drastically as taxon sampling increased, which contrasts the findings of Hamilton et al. (2014). This may be due to their use of maximum‐likelihood phylogenies that were converted to become ultrametric using the ‘chronopl’ and ‘multi2di’ functions in the R ‘ape’ package (Paradis et al., 2004). Talavera et al. (2013) found that this approach led to poorer performance in correctly identifying morphospecies and that if ML phylogenies are to be used, that PATHD8 (Britton et al., 2007) or r8s (Sanderson, 2003) software is more reliable. Other sources of this variation could be exacerbated by (1) sensitivity to intraspecific population structure, (2) an artefact of one or more violations of the GMYC model's assumptions, and (3) effects of incomplete lineage sorting or recent, rapid radiations within the group, or a combination of some or all of these factors.

The number of entities tended to exceed the estimated number of predefined groups in the full data sets (Table 1). This aligns with the conclusion made by Lohse (2009), in which the author stated that the ubiquity of population structure is likely to lead to the overestimation of meaningful species boundaries. This is a grey area in species delimitation, and users of the GMYC method should carefully define what ‘meaningful taxonomic units’ mean in the context of their study, particularly taking the frequency of singletons into account. It is a plausible hypothesis that the number of predefined groups is underestimations of true species diversity and that DNA‐based GMYC results are more accurate than traditional taxonomic classifications.

**TABLE 1:** SPEDE‐sampler results from the full datasets (100% sequence data) for the four case studies presented in the manuscript

	Cochineals	Tachinid flies	Fish	Ants
Gene	12S	COI	COI	COI
Number of sequences	142	736	741	267
Singletons (%)	3.33 ± 4.3	0	29.66 ± 0.25	52.9 ± 0.0
GMYC clusters	10.4 ± 0.52	30.8 ± 1.62	153.4 ± 0.7	65 ± 0.0
Max. GMYC clusters	11	34	155	65
Min. GMYC clusters	10	29	153	65
GMYC entities	10.8 ± 1.03	30.8 ± 1.62	218.1 ± 1.52	138 ± 0.0
Max. GMYC entities	12	34	221	138
Min. GMYC entities	10	29	216	138
User‐defined groups	5	20	194	88
GMYC exact matches, (%)	2, (40%)	15, (75%)	116, (60%)	29, (33%)
GMYC merges, (%)	0	2, (10%)	7, (4%)	7, (8%)
Match (+ singletons) (%)	100 ± 0.0	93.49 ± 0.33	96.74 ± 0.13	92.03 ± 0.0
Match (− singletons) (%)	100 ± 0.0	93.49 ± 0.33	95.37 ± 0.19	83.08 ± 0.0
SR (+ singletons)	2.16 ± 0.21	1.54 ± 0.08	1.12 ± 0.01	1.57 ± 0.0
SR (− singletons)	2.08 ± 0.1	1.54 ± 0.08	0.79 ± 0.0	0.74 ± 0.0

Note

Standard deviations are shown where appropriate.

Abbreviations: SR, splitting ratio; + singletons, including singletons; and − singletons, excluding singletons.

We showed across case studies how intraspecific geographical structuring could be mistaken for species‐level divergences and therefore inflated species richness estimates. Bergsten et al. (2012) showed how the identification success of barcode queries decreased as the geographical scale of sampling increased. This is a vital factor to consider in the sampling design and data analysis of species delimitation studies. The GMYC assumption of the absence of geographical substructuring within a data set is almost certainly violated in real‐world scenarios. Unbalanced sampling across distribution ranges may also contribute to variation in GMYC results, where data from different sampling scales may not always be comparable (Talavera et al., 2013).

Across cases, we could infer that the sampling carried out for the cochineal insects and tachinid flies had reached an asymptote and that further sampling is unlikely to yield greater species richness. The ants and the fish, however, displayed an increasing trajectory, suggesting that further sampling effort may result in the discovery of more diversity. These accumulation curves can be very useful to assist in future sample design, and to prioritize sampling effort in specific localities.

#### 3.3.2 Singletons

We found that the presence of singletons was generally associated with higher average splitting ratios and species richness estimates (i.e. the number of entities). The fish and ant case studies had the highest percentage of singletons (29.7% and 52.9% in the full data sets, respectively) and showed the largest differences between the number of clusters and entities, and between the splitting ratios including and excluding singletons (Table 1). Interestingly, these two case studies showed that the exclusion of singletons tended to result in merging by the GMYC (i.e. the GMYC merged groups that were believed to be separate by the user based on traditional taxonomy; Figures 4 and 5b).

Percentage match scores were generally not affected by the inclusion of singletons, with the exception of the ant case study, where singletons appeared to result in inflated estimates (Figure 5e and Table 1).

It is known that the GMYC model can accommodate a moderate number of singletons, but that skewed results have been observed when too many are included (Ahrens et al., 2016; Lim et al., 2012; Lohse, 2009; Puillandre et al., 2012). There are, however, contrasting reports in the literature regarding this effect. Talavera et al. (2013), for example, reported that although a higher proportion of singletons negatively affects biological meaningfulness, their GMYC success rate did not decrease even with a singleton incidence of 95%. Similarly, Ceccarelli et al. (2012) reported that despite their COI and cytochrome b data comprising 64% and 67% singletons, respectively, GMYC species richness estimates corroborated their morphological identifications. It is clear that the effects of singletons, and any other potential sampling effects, need to be assessed on a case‐by‐case basis. It is also important that other independent lines of evidence are acquired to complement single‐locus genetic data, such as additional genetic markers, geographical, behavioural and morphological information where applicable (Carstens et al., 2013).

## 4 CONCLUSION

The GMYC model is a very popular and widely applied tool in taxonomic and ecological contexts. We have developed SPEDE‐sampler as an open‐source software tool that offers insight into how computational and parameter choices, in combination with sampling effects, can influence GMYC output when applied to real‐world data sets. Factors including the proportion of singletons present, sample size and geographical collection coverage, and intraspecific population structuring can have significant effects on species delimitation estimates. Additionally, through comparing the number of GMYC species estimates with user‐predefined groups, SPEDE‐sampler can assist users in identifying which groups are not as diverse as previously thought, and which may contain cryptic species or undiscovered diversity. These can then be prioritized for further studies (e.g. interbreeding and hybridization, taxonomy). The examples presented here have illustrated the workflow and functionality of SPEDE‐sampler across different taxa and data set sizes, and have highlighted the importance of interpreting the output contextually.

## CONFLICT OF INTEREST

The authors declare that they have no known competing financial interests or personal relationships that could have appeared to influence the work reported in this study.

## AUTHOR CONTRIBUTIONS

Clarke van Steenderen conceptualized the study, performed formal analysis, contributed to methodology, software writing and software validation, wrote the original draft, and wrote, reviewed and edited the manuscript. Guy Sutton conceptualized the study, wrote the original draft, and wrote, reviewed and edited the manuscript.

## Supporting information

Data S1

Data S2

Data S3

Data S4

Data S5

Data S6

Data S7

Data S8

Data S9

Data S10

Data S11

Data S12

Data S13

Figure S1

## Data Availability

The software code for SPEDE‐sampler is freely available on GitHub, and the data that support the findings of this study are available on Google Drive.
