# Supplementary figures and images for: SPEDE‐sampler: An R Shiny application to assess how methodological choices and taxon sampling can affect Generalized Mixed Yule Coalescent output and interpretation
Source: Mol Ecol Resour. 2022 Feb 16;22(5):2054–69. doi: 10.1111/1755-0998.13591 (PMC9306842; doi:10.1111/1755-0998.13591)

SPEDE-SAMPLER R SHINY GUI INTERFACE

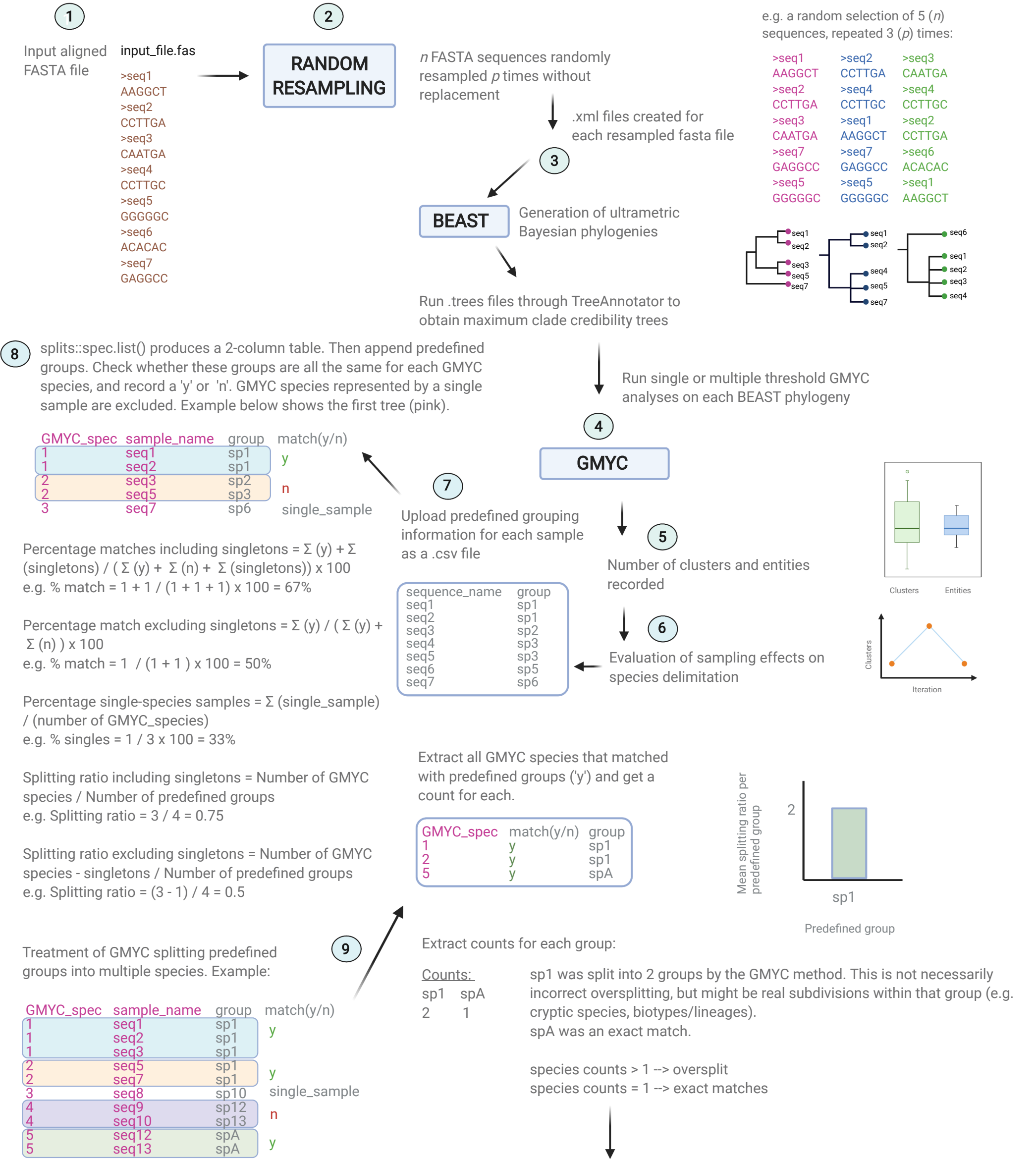

Supplement: Supplementary file 14 — Figure S1 [file MEN-22-2054-s011.csv]
